# Lipo-oxytocin-1, a Novel Oxytocin Analog Conjugated with Two Palmitoyl Groups, Has Long-Lasting Effects on Anxiety-Related Behavior and Social Avoidance in *CD157* Knockout Mice

**DOI:** 10.3390/brainsci5010003

**Published:** 2015-01-20

**Authors:** Akira Mizuno, Stanislav M. Cherepanov, Yusuke Kikuchi, Azam AKM Fakhrul, Shirin Akther, Kisaburo Deguchi, Toru Yoshihara, Katsuhiko Ishihara, Satoshi Shuto, Haruhiro Higashida

**Affiliations:** 1 Faculty of Pharmaceutical Sciences, Hokkaido University, Kita-12, Nishi-6, Kita-ku, Sapporo 060-0812, Japan; E-Mails: nrh23255@gmail.com (A.M.); kikuchiym@ec.hokudai.ac.jp (Y.K.); 2 Department of Basic Research on Social Recognition, Research Center for Child Mental Development, Kanazawa University, Kanazawa 920-8640, Japan; E-Mails: stas4476@mail.ru (S.M.C.); akmfazam@yahoo.com (A.A.F.); akthershirin1182@yahoo.com (S.A.); toruyosi@kiea.m.kanazawa-u.ac.jp (T.Y.); 3 Medical Research Institute, Kanazawa Medical University and Medical Care Proteomics Biotechnology Co., Uchinada, Ishikawa 920-0293, Japan; E-Mail: kdeguchi@kanazawa-med.ac.jp; 4 Department of Immunology and Molecular Genetics, Kawasaki Medical School, Kurashiki, Okayama 701-0192, Japan; E-Mail: ishihara-im@med.kawasaki-m.ac.jp; 5 Center for Research and Education on Drug Discovery, Hokkaido University, Kita-12, Nishi-6, Kita-ku, Sapporo 060-0812, Japan

**Keywords:** synthetic oxytocin, oxytocin analog, lipidation, social behavior, social avoidance, autism, *Bst1*, *CD157*

## Abstract

Oxytocin (OT) is a nonapeptide hormone that is secreted into the brain and blood circulation. OT has not only classical neurohormonal roles in uterine contraction and milk ejection during the reproductive phase in females, but has also been shown to have new pivotal neuromodulatory roles in social recognition and interaction in both genders. A single administration of OT through nasal spray increases mutual recognition and trust in healthy subjects and psychiatric patients, suggesting that OT is a potential therapeutic drug for autism spectrum disorders, schizophrenia, and some other psychiatric disorders. Although the mechanism is not well understood, it is likely that OT can be transported into the brain where it activates OT receptors to exert its function in the brain. However, the amount transported into the brain may be low. To ensure equivalent effects, an OT analog with long-lasting and effective blood-brain barrier penetration properties would be beneficial for use as a therapeutic drug. Here, we designed and synthesized a new oxytocin analog, lipo-oxytocin-1 (LOT-1), in which two palmitoyl groups are conjugated at the amino group of the cysteine9 residue and the phenolic hydroxyl group of the tyrosine8 residue of the OT molecule. To determine whether LOT-1 actually has an effect on the central nervous system, we examined its effects in a *CD157* knockout model mouse of the non-motor psychiatric symptoms of Parkinson’s disease. Similar to OT, this analog rescued anxiety-like behavior and social avoidance in the open field test with the social target in a central arena 30 min after intraperitoneal injection in *CD157* knockout mice. When examined 24 h after injection, the mice treated with LOT-1 displayed more recovery than those given OT. The results suggest that LOT-1 has a functional advantage in recovery of social behavioral impairment, such as those caused by neurodegenerative diseases, autism spectrum disorders, and schizophrenia.

## 1. Introduction

**Figure 1:**
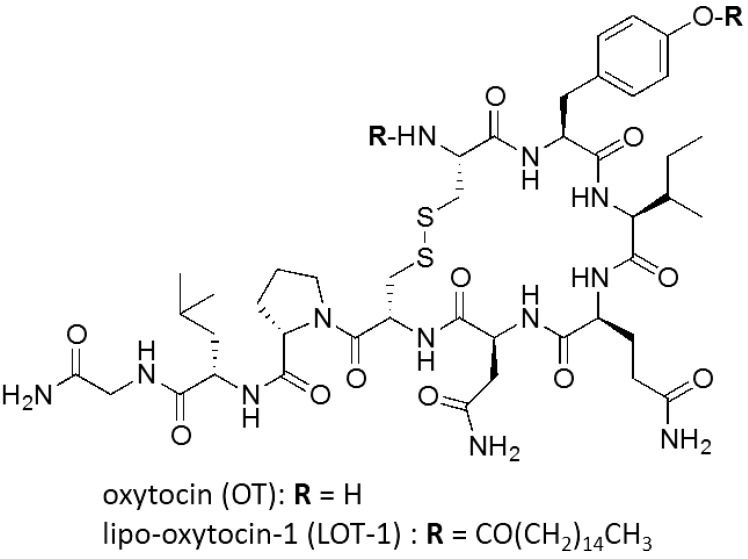
Chemical structures of oxytocin (OT) and its analog, lipo-oxytocin-1 (LOT-1).

Autism spectrum disorders (ASDs) are neuropsychiatric conditions characterized by impairments in social interactions, social communication, and the presence of repetitive behaviors and restricted interests, with or without associated intellectual disability [1]. Although many clinicians and psychotherapists have attempted various psychotropic medications and psychoeducational treatments [2], there is still no therapeutic strategy for social impairments in patients with ASDs [3]. Recently, a great deal of research has focused on oxytocin (OT), a cyclic nonapeptide, the structure of which is shown in Figure 1. OT is secreted into the brain from oxytocin-synthesizing (oxytocinergic) neurons in the hypothalamus where it functions as a neurotransmitter and/or neuromodulator [4,5] and enters the blood circulation from the nerve endings of oxytocinergic neurons in the posterior pituitary as a hormone [4,5,6,7,8]. Various rodent studies indicated that OT plays an important role in social recognition and memory [9,10] in social preference and interactions [11] as well as in social fear [12]. Moreover, in human studies, intranasal OT resulted in improvement in feelings of trust, the ability to infer the mental state of others, and time spent gazing toward the eye region in rodents and humans [5,7,8,9,10,11,12,13,14,15,16,17,18,19], which are frequent deficits in ASD patients [10,18,20,21,22]. Considering the above results obtained with a single administration, OT is expected to be an effective drug for the treatment of social impairment in patients with psychiatric disorders, such as ASDs and schizophrenia [7,10,15,18,20,21,22,23,24,25]. However, clinical trials with OT have only begun recently, and its effectiveness has not yet been established, with many questions remaining to be answered [18,20,23]. One question is whether the administered OT indeed functions in the central nervous system (CNS) as a drug [14] given its short lifetime in the blood, with a half-life of 2.4–20 min [14,26,27] and its poor brain delivery profile [27,28,29]. Therefore, we planned to develop OT analogs, with greater efficacy for ASD treatment than OT itself due to the enhanced pharmacokinetic properties introduced by chemical modification.

Bioactive peptides, mostly peptide hormones, have been used effectively for clinical disease therapy [30]. The therapeutic use of natural peptide hormones is, however, substantially limited due to undesired pharmacokinetic properties, such as poor absorption and brain transport, low metabolic stability, and rapid excretion [30,31]. Many methodologies have been developed to address the undesired pharmacokinetic properties of natural peptide hormones [30,31]. One successful methodology for this purpose, especially for elongation of the half-life in blood, is lipidation, which involves conjugating a peptide hormone with a long fatty acid [32].

Although effects of lipidation on blood-brain barrier (BBB) penetration of the parent molecule are poorly documented, it is well known that hydrophobic small molecules, *i.e.*, those with a high Log*P* value, are more likely to penetrate the BBB than hydrophilic molecules [30]. Therefore, we thus hypothesized that the lipidation strategy would effectively enhance the hydrophobicity of OT and therefore not only extend its half-life in the blood but also improve its brain delivery profile. Based on this hypothesis, we designed and synthesized an OT analog, lipo-oxytocin-1 (LOT-1), in which two palmitoyl groups are conjugated at the amino group of the Cys 9 residue and the phenolic hydroxyl group of the Tyr 8 residue, as shown in Figure 1.

Here, we evaluated effects of the new OT analog on impairment of social behavior in the newly developed CD157 knockout (*CD157^−/−^*) mouse strain [33] to determine whether LOT-1 is effective in the CNS. *CD157*^−/−^ mice display severe psychiatric features with anxiety-related and depression-like behaviors, fear, and social avoidance, which were originally considered psychiatric non-motor symptoms of Parkinson’s disease [33]. Furthermore, we also showed that this social impairment in *CD157*^−/−^ mice can be readily recovered by a single injection of OT [33], suggesting that such phenotypes may be shared with other psychiatric impairments, such as ASDs and schizophrenia. Here, we report that LOT-1 has an advantage over native OT with respect to long-lasting *in vivo* effects, while OT has better effects than this analog on recovery of social impairment shortly after treatment.

## 2. Results

### 2.1. Compound Synthesis

We synthesized the new OT analog, lipo-oxytocin-1 (LOT-1), the structure of which is shown in Figure 1, as follows. Palmitic anhydride (25 mg, 51 μmol) in CH_2_Cl_2_ (0.80 mL) was added to a solution of oxytocin acetate (42 mg, 39 μmol) and triethylamine (17 μL, 0.12 mmol) in DMF (0.80 mL), and the resulting mixture was stirred for three hours. After removal of the solvent under reduced pressure, the residue was washed with Et_2_O, filtered, and dried to give *N*-palmitoyloxytocin as a white powder (46 mg). Palmitoyl chloride (17 μL, 56 mmol) in CH_2_Cl_2_ (1.6 mL) was added to a solution of the obtained *N*-palmitoyloxytocin white powder (46 mg), triethylamine (15 μL, 110 μmol) and *N*,*N-*dimethylaminopyridine (3.6 mg, 30 μmol) in DMF (1.6 mL), and the resulting mixture was stirred overnight. After the reaction was quenched by addition of MeOH, the solvent was removed under reduced pressure. The residue was washed with Et_2_O and purified by silica gel column chromatography (silica gel supporting COOH, 50%–100% EtOH in AcOEt) to give LOT-1 (44 mg, 30 μmol, 79%) as a white powder: ^1^H-NMR (400 MHz, DMSO-*d*_6_) d 8.80 (1 H, br), 8.31–8.22 (3 H, m), 8.12 (1 H, d), 7.97–7.90 (2 H, m), 7.49 (1 H, br), 7.41–7.27 (4 H, m), 7.10 (1 H, s), 7.06 (1 H, s), 6.95 (2 H, d), 6.88 (1 H, s), 6.81 (1 H, s), 5.05 (1 H, br), 4.76–4.62 (2 H, m), 4.40 (1 H, m), 4.32 (1 H, m), 4.16 (1 H, m), 3.97 (1 H, br), 3.86 (1 H, br), 3.68–3.48 (4 H, m), 3.24 (1 H, m), 3.06–2.78 (4 H, m), 2.70–2.50 (4 H, m), 2.31 (2 H, s), 2.16–1.70 (10 H, m), 1.70–1.04 (57 H, m), 0.98–0.72 (18 H, m); HRMS (ESI) calculated for C_75_H_126_N_12_O_14_ S_2_Na: 1505.8850 [(M + Na)^+^], found: 1505.8807. The molecular weight is 1483. Purity of LOT-1 was determined as 98.2% by HPLC (Kinetex 1.7 μ C8, 2.1 × 50 mm; A 0.05% aq. Formic acid, B 0.05% formic acid in MeCN; 1–10 min/1%–95% B gradient, 10–20 min/95% B; retention time 9.9 min), where OT (retention time 2.6 min) was below the detection level.

### 2.2. Anxiety Behavior

**Figure 2:**
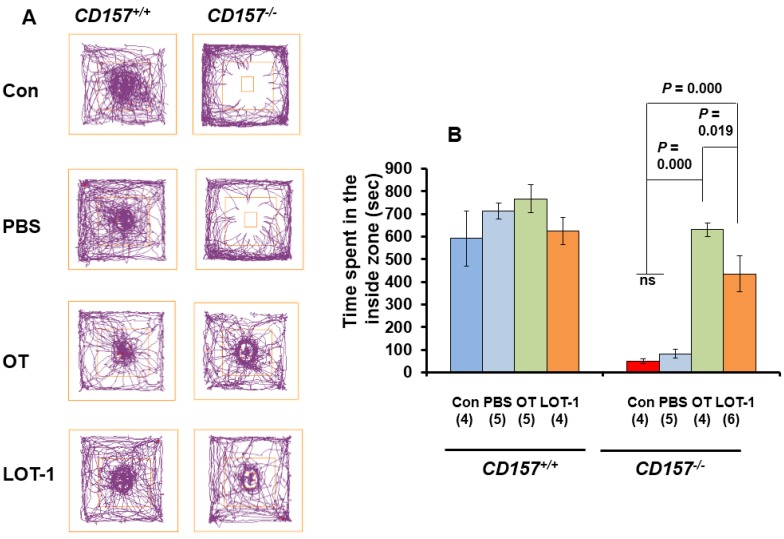
Anxiety and social preference tests 30 min after injection of OT or LOT-1. (**A**) Representative traces show movement tracks of the *CD157*^+/+^ and *CD157*^−/−^ mice over a 20-min period in the social-interaction test in the open field with the social target in the center. Adult male mice were treated with or without intraperitoneal injection of phosphate buffered saline (PBS), OT (100 ng/kg of body weight), or LOT-1 (100 ng/kg of body weight) 30 min prior the behavioral experiments. A male mouse as a social target was placed in the center immediately after a 10-min habituation period; (**B**) Time spent in the inside zone measured over 20 min in *CD157*^+/+^ and *CD157*^−/−^ mice with different treatments. The data are expressed as the means ± s.e.m. The number of mice is in each experimental group is shown in parentheses. One-way ANOVA in one genotype: for *CD157*^+/+^ mice (2B, left), *F*_3,14_ = 1.01, *P* < 0.4188; for *CD157*^−/−^ mice (2B, right), respectively. Two-way ANOVA for genotypes (*CD157*^+/+^ and *CD157*^−/−^) and drugs (PBS/OT/LOT-1): *F*_3,37_ = 9.22, *P* < 0.0002.

When mice were exposed to the novel environment in the open-field apparatus with a social target (a male mouse) in the center, judging from the observed tracks traveled, *CD157*^−/−^ male mice engaged in less exploratory behavior, particularly in the inside zone, than *CD157^+/+^* males, as shown in representative traces (Figure 2A, top). This suggests that *CD157*^−/−^ mice displayed anxiety-like behavior and social avoidance behavior, while *CD157^+/+^* mice showed intensive interaction with the target mouse. This behavioral feature was unchanged 30 min after intraperitoneal injection of saline (PBS) in both genotypes (Figure 2A, second row). A marked change was elicited by intraperitoneal OT injection (100 ng/kg body weight) in *CD157*^−/−^ mice, but no change was observed in *CD157^+/+^* mice (Figure 2A, third row). Injection of LOT-1 (100 ng/kg body weight) resulted in essentially similar recovery from social impairment, while no additional or specific effect was induced by LOT-1 in *CD157^+/+^* mice (Figure 2A, bottom). One-way ANOVA revealed no significant difference in *CD157^+/+^* mice (*n* = 4–5, *F*_3,14_ = 1.01, *P* = 0.4188) but a significant difference in *CD157*^−/−^ mice (*n* = 4–6, *F*_3,15_ = 54.14, *P* = 0.0000) (Figure 2B). Bonferroni’s *post hoc* tests in *CD157*^−/−^ mice indicated *P* = 0.0000 between both non- or saline-treated controls and OT; *P* = 0.0000 between both controls and LOT-1; and *P* = 0.019 between OT- and LOT-1-treated mice. Two-way ANOVA analysis for genotypes and drugs resulted in *F*_37,128_ = 9.22, *P* = 0.0002. The results indicated that the synthetic analog, LOT-1, had a significant effect on recovery of social behavior, but the effect seems to be lower than that of native OT at the 30-min time point after treatment.

**Figure 3:**
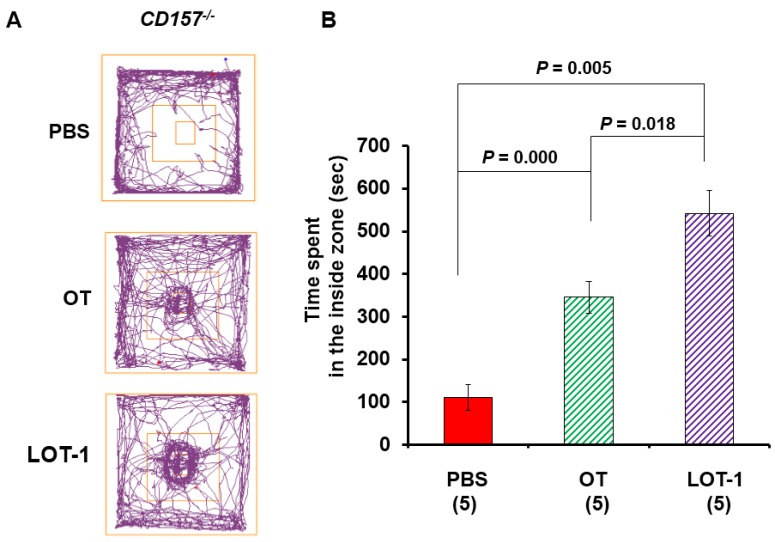
Anxiety and social preference tests 24 h after injection of OT or LOT-1. (**A**) Representative traces show movement tracks of the *CD157*^−/−^ mice over a 20-min period in the social-interaction test in the open field with the social target in the center. Adult male mice were treated with intraperitoneal injection of phosphate buffered saline (PBS), OT (100 ng/kg of body weight), or LOT-1 (100 ng/kg of body weight), one day (24 h) prior to the behavioral experiments; (**B**) Time spent in the inside zone measured over 20 min in *CD157*^−/−^ mice with different treatments. The data are expressed as the means ± s.e.m. The number of mice in each experimental group is shown in parentheses. One-way ANOVA for drugs: *F*_2,12_ = 27.05, *P* < 0.0001. Bonferroni’s *post hoc* test indicated between PBS and OT (*P* = 0.0000), PBS and LOT-1 (*P* = 0.005), and OT and LOT-1 (*P* = 0.018).

To determine whether this anti-anxiety effect of OT and/or LOT-1 in *CD157*^−/−^ mice has long-lasting effects (Figure 3A), we tested the time-dependency of the effects on both parameters in the inside zone 24 h after treatment (Figure 3). Interestingly, LOT-1 treatment retained the rescue effect to a greater extent than OT (one-way ANOVA, *F*_2,12_ = 27.05, *P* = 0.0000). As shown in Figure 3B, Bonferroni’s *post hoc* test indicated significant differences between PBS and OT (*P* = 0.0000), PBS and LOT-1 (*P* = 0.005), or OT and LOT-1 (*P* = 0.018) (*n* = 5 per group).

## 3. Discussion

Here, we applied the lipidation strategy [28] to improve the drug-like properties of OT. Two palmitoyl groups were conjugated to the OT molecule, and the resultant molecule was designated as lipo-oxytocin-1 (LOT-1). The results showed that 30 min after injection into *CD157* knockout mice, LOT-1 had slightly less effect than synthetic native OT on interaction with the social target (a mouse of the same sex) in the central zone in the open-field test arena. In these experiments, the same doses (100 ng/kg of body weight) of OT and LOT-1 were injected into the mice; however, the molecular weight of LOT-1 (mw = 1483) is approximately 1.5 times higher than that of OT (mw = 1007). This suggested that anxiety-like behavior and social avoidance were significantly improved by the analog with approximately the same potency. Interestingly, however, LOT-1 had a greater effect on rescue the next day (24 h after drug injection), suggesting that LOT-1 could have a longer half-life and may have a long-lasting effect superior to native OT, even though the molar dose of LOT-1 was less than that of OT. Thus, LOT-1 is of vital importance as a potent and durable OT analog that is likely to have a significant advantage over OT itself in therapy for psychiatric disorders such as ASDs.

In the present study, we directly measured the effects of the analog *in vivo*, bypassing many *in vitro* experiments. In addition, we used unique *CD157*^−/−^ mice, which were characterized very recently [33]. This mouse line displays severe anxiety-like and depression-like behavior when well-established behavior monitoring devices were applied. *CD157* was first isolated as bone marrow stromal cell antigen-1 [34], and later, several single nucleotide polymorphisms (SNPs) of the *CD157* gene were reported to be associated with Parkinson’s disease [35]. As we did not detect any motor dysfunction in the knockout mice, although spontaneous motor activities in daily life were very low, we hypothesized that *CD157* SNPs are risk alleles for psychiatric symptoms rather than motor impairments, both of which are hallmarks of Parkinson’s disease.

Lipidation is a peptide modification that can effectively develop peptide drugs with long-lasting activity [32]. For example, an insulin analog conjugated with hexadecanoic acid (insulin degludec) has a duration of action that lasts more than 42 h and is called an ultralong-acting insulin analog [36]. Another lipidated peptide, a glucagon-like peptide-1 analog conjugated with palmitic acid (liraglutide), is now clinically used for the treatment of type 2 diabetes mellitus and has a long plasma half-life of 10–12 h [37]. To our knowledge, however, there have been no reports regarding lipidated OT, and therefore, LOT-1 is the first effective lipid-modified oxytocin.

OT applied peripherally has been shown to exert various effects on socioemotional behavior, which may be partly due to peripheral effects (e.g., mediated by sympathetic nervous system), as described [38,39]. However, we assumed that LOT-1 was recruited into the brain, as showed previously that cerebrospinal OT concentration was increased after subcutaneous injection of OT into ICR mice [5]. However, there is no evidence of efficient OT or LOT-1 uptake into the brain. It would be important to show that the novel agonist is indeed taken up into the brain via the BBB.

## 4. Experimental Section

### 4.1. Animals

*CD157*^−/−^ mice were maintained by crossbreeding homozygous mutant mice [33,34]. C57BL/6 wild-type (*CD157^+/+^*) and *CD157*^−/−^ mice were kept in the animal center under standard conditions (22 °C; 12-h light/dark cycle, lights on at 08:45 a.m.) in standard mouse cages (300 mm × 160 mm × 110 mm) with sawdust as bedding and received food and water *ad libitum*, as described previously [5]. Breeding pairs were maintained separately (1 pair per cage). All animal experiments were carried out in accordance with the Fundamental Guidelines for Proper Conduct of Animal Experiment and Related Activities in Academic Research Institutions under the jurisdiction of the Ministry of Education, Culture, Sports, Science and Technology of Japan, and were approved by the Committee on Animal Experimentation of Kanazawa University.

### 4.2. Anxiety-Like Behavior and Social Avoidance Test

Anxiety-like behavior in the unfamiliar open-field and social-avoidance behavior toward a novel target (wild-type C57BL/6) male mouse were measured in a two-stage social-interaction test [40] as described previously [33]. This is a mix of an open field test described for non-social anxiety and a social preference test [10,11,41]. Briefly, in the first 10-min test (target absent, adaptation phase), the experimental mouse was allowed to freely explore a square-shaped arena (600 mm × 600 mm) containing a wire mesh cage (70 mm × 90 mm × 70 mm and bars spaced 5 mm apart) placed in the center of the arena. In the second 20-min test, the experimental mouse was reintroduced into the arena with an unfamiliar C57BL/6 male mouse in a wire mesh cage. Video tracking software (ANY-maze; Stoelting Co., Wood Dale, IL, USA) was used to measure the amount of time the experimental mouse spent in the “interaction zone” (300 mm × 300 mm). Behavior was examined in mice at 20 min and 24 h after intraperitoneal injection of 10 ng/mL OT or LOT-1 (approximately 100 ng/kg body weight) or phosphate buffered saline (PBS) or without any treatment.

### 4.3. Statistical Analysis

The data were calculated as the means ± s.e.m., and analyzed by two-way ANOVA or one-way ANOVA followed by Bonferroni’s *post hoc* test. In all analyses, *P* < 0.05 was taken to indicate statistical significance.

## 5. Conclusions

Here, as we have demonstrated, the *in vivo* effect and the anxiolytic potential of LOT-1, lipidation is an appropriate approach for the generation of new therapeutic OT derivatives. However, the affinity to OT receptors in OT receptor expressing cells [42], membrane permeability, and efficacy in transportation across the BBB of LOT-1 remain to be determined. It will also be necessary to determine how much the analog stimulates OT receptors and the stability of the peptide analog against enzymatic degradation or cleavage. These experiments are currently underway in our laboratory. In addition, it may be necessary to measure the concentrations of LOT-1 and OT, which would represent the natural OT and OT derived from LOT-1, in the cerebrospinal fluid.
